# Protective Role of 4-Octyl Itaconate in Murine LPS/D-GalN-Induced Acute Liver Failure via Inhibiting Inflammation, Oxidative Stress, and Apoptosis

**DOI:** 10.1155/2021/9932099

**Published:** 2021-08-17

**Authors:** Ruidong Li, Wenchang Yang, Yuping Yin, Peng Zhang, Yaxin Wang, Kaixiong Tao

**Affiliations:** ^1^Department of Gastrointestinal Surgery, Union Hospital, Tongji Medical College, Huazhong University of Science and Technology, Wuhan 430022, China; ^2^Department of Critical Care Medicine, Union Hospital, Tongji Medical College, Huazhong University of Science and Technology, Wuhan 430022, China

## Abstract

Oxidative stress, inflammation, and apoptosis are crucial in the pathogenesis of acute liver failure (ALF). 4-Octyl itaconate (OI) showed antioxidative and anti-inflammatory properties in many disease models. However, its role in lipopolysaccharide- (LPS-)/D-galactosamine- (D-GalN-) induced ALF is still not investigated. Here, we established an ALF murine model induced by LPS/D-GalN administration. And we found that OI improved survival rate in the murine ALF model. Our results also showed that OI alleviated LPS/D-GalN-induced hepatic histopathological injury and reduced the serum activities of alanine transaminase and aspartate transaminase. Moreover, OI reduced serum levels of proinflammatory cytokines such as monocyte chemotactic protein-1, tumor necrosis factors-*α*, and interlukin-6. Additionally, OI mitigated oxidative stress and alleviated lipid peroxidation in a murine model of ALF. This was evaluated by a reduction of thiobarbituric acid reactive substances (TBARS) in liver tissues. In addition, OI increased the ratio of reduced glutathione/oxidized glutathione and the activities of antioxidant enzymes including catalase and superoxide dismutase. Moreover, the apoptosis of hepatocytes in the liver was inhibited by OI. Furthermore, we found that OI inhibited LPS-induced nuclear translocation and activation of factor-kappa B (NF-*κ*B) p65 in macrophages which could be inhibited by OI-induced activation of nuclear factor erythroid-2-related factor (Nrf2) signaling. Additionally, D-GalN-induced reactive oxygen species (ROS) generation and apoptosis in hepatocytes were inhibited by OI-induced activation of Nrf2 signaling. Therefore, the underlying mechanism for OI's protective effect in LPS/D-GalN-induced ALF may be associated with deactivation of NF-*κ*B signaling in macrophages to reduce inflammation and inhibition of ROS-related hepatocyte apoptosis by activating Nrf2. In conclusion, OI showed a protective role in LPS/D-GalN-induced ALF by reducing inflammation, enhancing antioxidant capacity, and inhibiting cell apoptosis.

## 1. Introduction

Acute liver failure (ALF) is a critical clinical syndrome that leads to high mortality in clinic [1]. Pathogenic factors such as viral infection, toxic chemicals, and hepatotoxic drugs can cause ALF [2–5]. Lipopolysaccharide (LPS) is a component of the outer cell wall of gram-negative bacteria, which can induce liver damage by activating inflammatory cytokine production [6]. D-Galactosamine (D-GalN) is a hexosamine derived from galactose which can cause hepatic damage [7]. LPS and D-GalN can lead to hepatic injury in the mouse, which is similar to fulminant hepatic failure in clinic [8]. Therefore, a LPS/D-GalN-induced hepatic injury model has been widely used to explore potential hepatoprotective medicines. It has been widely admitted that excessive oxidative stress resulting from reactive oxygen species (ROS) and hepatocyte apoptosis are two pivotal factors attributed to LPS/D-GalN-induced liver injury [9, 10]. Normally, hepatocytes are protected from oxidative injury by antioxidative enzymes in the liver. Many antioxidative genes are regulated by Nrf2, and activation of Nrf2 shows a protective effect in hepatic diseases [5, 11, 12]. Normally, Nrf2 is combined with Kelch-like ECH2-associated protein 1 (Keap1) in the cytoplasm. In response to oxidative stress, Nrf2 could dislocate from Keap1 and translocate into the nucleus and then activate antioxidative gene transcription against oxidative damage [13, 14]. Apart from oxidative stress, excessive inflammatory response has been confirmed as an important pathogenic factor in LPS/D-GalN-induced ALF. Nuclear factor-*κ*B (NF-*κ*B) is an important nuclear transcription factor and involved in inflammatory response [15, 16]. Under the stimulus of LPS/D-GalN, NF-*κ*B was activated and increased the production of proinflammatory cytokines such as TNF- (tumor necrosis factor-) *α*, interleukin- (IL-) 6, and IL-1*β* [17]. Excessive proinflammatory cytokines are associated with hepatic cell injury.

Macrophage activation is pivotal in the inflammatory response and cellular metabolism. Inflammatory stimuli alter the status of respiration and tricarboxylic acid (TCA) cycle, which leads to the accumulation of endogenous metabolites. Itaconate is one of these metabolites capable of regulating inflammation [18]. Itaconate is synthesized by *cis*-aconitate decarboxylase (CAD). Citrate is transformed to *cis*-aconitate by aconitate hydratase 2. Then, *cis*-aconitate is catalyzed by CAD and generates itaconate. Itaconate is highly induced in activated macrophages and shows remarkable ability in inhibiting inflammatory response [19]. Mechanically, itaconate exerts controlling of inflammation and oxidative stress by competitive inhibition of succinate dehydrogenase (SDH) mediated oxidation and degradation of Kelch-like ECH-associated protein 1 (Keap1) to activate the Nrf2 pathway [18, 19]. Accordingly, itaconate and its derivates, such as 4-octyl itaconate (OI) and dimethyl-itaconate, showed protective effects in many inflammatory diseases' models [12, 20]. However, their functional role in LPS/D-GalN-induced ALF is still unclear.

In our study, we explored the effect of OI in LPS/D-GalN-induced ALF murine model. Our results showed that OI mitigated LPS/D-GalN-induced liver injury and improved survival rate. OI could inhibit oxidative stress and inflammatory response in a LPS/D-GalN-induced ALF model. The possible mechanism was attributed to the inhibition of NF-*κ*B activation and promoting Nrf2 signaling. Our results therefore highlight OI as a potential therapeutic reagent for the treatment of ALF.

## 2. Materials and Methods

### 2.1. Reagents and Antibodies

Fetal bovine serum, phosphate-buffered saline (PBS), and high-glucose Dulbecco's modified Eagle's medium (DMEM) were purchased from Gibco Life Technologies (Carlsbad, CA, USA). The alanine transaminase (ALT), catalase (CAT), aspartate transaminase (AST), reduced glutathione/oxidized glutathione (GSH/GSSG), myeloperoxidase (MPO), and superoxide dismutase (SOD) assay kit were obtained from Nanjing Jiancheng Institute of Biotechnology (Nanjing, China). The Cell Counting Kit-8 kit, 2,7-Dichlorofluorescein diacetate (DCFH-DA), TUNEL apoptosis assay kit, and BeyoECL Plus were bought from Beyotime Biotechnology (Shanghai, China). OI was purchased from MedChemExpress (USA). LPS and D-GalN were purchased from Sigma-Aldrich (St. Louis, MO, USA). The enzyme-linked immunosorbent assay (ELISA) kits for detecting mouse TNF-*α*, IL-6, and MCP-1 (monocyte chemotactic protein-1) were obtained from Dakewe Bioengineering (Shenzhen, China). The antibodies against Bax, Bcl-2, Nrf2, NF-*κ*B p65, cleaved poly-ADp ribose polymerase (PARP), phosphor-NF-*κ*B p65, and I*κ*B-*α* were obtained from Cell Signaling Technology (Beverly, MA, USA). Antiglyceraldehyde 3-phosphate dehydrogenase (GAPDH) antibodies were purchased from Proteintech (Wuhan, China). All other chemicals and solvents used were of the highest analytical grade.

### 2.2. Animal and Experiment Design

Male C57BL/6 mice (23–25 g) were bought from SPF Biotechnology Co., Ltd. (Beijing, China). The animal experiment was approved by the Animal Care and Use Committee of Tongji Medical College of Huazhong University of Science and Technology. The animals were raised according to the National Health Guidelines for the Care and Use of Laboratory Animals. ALF model was performed as described previously with minor alteration [8]. To determine a suitable dose for the next experiment, mice were divided into 5 groups (*n* = 6 to 12/per group). Then, mice were given different doses of LPS/D-GalN: LPS (0 mg/kg)/D-GalN (0 mg/kg), LPS (1 mg/kg)/D-GalN (200 mg/kg), LPS (1 mg/kg)/D-GalN (300 mg/kg), LPS (1 mg/kg)/D-GalN (400 mg/kg), and LPS (1 mg/kg)/D-GalN (500 mg/kg). Once a suitable dose was chosen, mice were divided into 4 groups (*n* = 5/per group): (1) control group: mice received the same volume vehicle during the experiment; (2) OI group: mice were given OI without LPS/D-GalN; (3) LPS/D-GalN group: mice received LPS/D-GalN without OI; and (4) LPS/D-GalN+OI group: mice received LPS/D-GalN and OI. OI was dissolved in PBS (concentration: 2 mg/ml) and then was given by intraperitoneal injection (50 mg/kg body weight) 2 h before LPS/D-GalN or vehicle treatment. LPS/D-GalN was given in a final volume of 200 *μ*l by intraperitoneal injection. The tissue samples and serum were collected and analyzed at the according time point in the next experiment.

### 2.3. Liver Histopathology and Immunohistochemistry

Hepatic tissues were collected 6 h after LPS/D-GalN administration and then were fixed in 4% paraformaldehyde solution at 4°C overnight. Then, the fixed tissue portions were processed into paraffin blocks. 4 *μ*m thick sections were cut and mounted on glass slides. Then, sections were stained with hematoxylin and eosin (H&E). Hepatic damage was graded as described previously [21]. Briefly, liver necrosis, congestion, and degeneration were graded as follows: 0: absent; 1: mild; 2: moderate; and 3: severe. For immunohistochemistry, 4 *μ*m thick murine paraffin-embedded hepatic sections were stained with myeloperoxidase primary antibody (Santa Cruz, CA, USA) and visualized by the appropriate HRP-conjugated secondary antibody and 3,3-diaminobenzidine tetrahydrochloride.

### 2.4. ALT and AST Assay

Serum was collected 6 h after LPS/D-GalN administration. Then, ALT and AST activities were measured according to the manufacturer's instructions (Nanjing Jiancheng Biological Technology, Nanjing, China).

### 2.5. The Cell Culture and Treatment

The murine macrophage cell line RAW264.7 was bought from the Cell Bank of the Chinese Academy of Sciences (Shanghai, China). The murine normal hepatic cell line NCTC1469 was obtained from China Center for Type Culture Collection (Wuhan, China). Both cell lines were cultured in Dulbecco's modified Eagle's medium (DMEM, high glucose) with 10% fetal bovine serum under a humidified atmosphere of 5% CO_2_ at 37°C. RAW264.7 cells (2∗10^5^ per well) were seeded in 6-well plates overnight. Then, OI (100 *μ*M) or vehicle was added 2 h before LPS (200 *μ*g/ml) administration. Then, cells were collected for western blot or immunofluorescence 6 h later. For measurement of cell viability, NCTC 1469 cells (1∗10^4^ per well) were seeded in 96-well plates overnight. Different concentrations of D-GalN (10 mM, 20 mM, 30 mM, and 40 mM) or OI (10 *μ*M, 50 *μ*M, and 100 *μ*M) were added, respectively, for 12 h to determine a suitable concentration for the next experiments. Then, NCTC 1469 cells (2∗10^5^ per well) were seeded in 6-well plates overnight. OI (100 *μ*M) was added 2 h before D-GalN (40 mM) treatment. After D-GalN was given for 12 h, cells were collected for western blot, ROS assay, and Annexin V/7-amino-actinomycin D staining.

### 2.6. Cell Viability Assay

Cell Counting Kit-8 kit was used to determine the effect of OI or/and D-GalN on NCTC 1469 cells according to the manufacturer's instructions. Briefly, cells (1∗10^4^ per well) were grown in 96-well plates overnight. After interference, 10 *μ*l CCK-8 solution was added and incubated for 2 h. Then, the absorbance was measured at 450 nm on a microplate reader and cell viability was calculated by optical density. The cell viability of the control group was defined as 100%.

### 2.7. Measurement of TBARS, CAT, SOD, and GSH/GSSG Ratio in the Liver

Briefly, hepatic tissues were homogenized in iced-cold PBS (weight/volume = 1 : 10). Then, the homogenized tissues were centrifuged at 10000 × g (4°C) for 15 minutes. The supernatants were collected and used for TBARS, CAT, SOD, and GSH/GSSG evaluation by a commercial detection kit (Nanjing Jiancheng Institute of Biotechnology, Nanjing, China) according to the manufacturer's instructions.

### 2.8. Cytokine Activity by ELISA and Hepatic MPO Activity

The levels of IL-6, TNF-*α*, and MCP-1 at 2 h, 4 h, and 6 h after LPS/D-GalN administration in serum were measured using the respective ELISA assay kits according to the manufacturer's instructions (Dakewe Bioengineering, Shenzhen, China). MPO activity was measured using a commercial detection kit (Nanjing Jiancheng Institute of Biotechnology, Nanjing, China) according to the manufacturer's instructions.

### 2.9. The Detection of Hepatocyte Apoptosis

Terminal deoxynucleotidyl transferase-mediated dUTP nick-end labeling (TUNEL) method was used to evaluate hepatocyte apoptosis. Briefly, the waxed sections were dewaxed and rehydrated using xylene and a graded series of ethanol and then were administrated with 20 *μ*l/ml proteinase K without DNase for 35 minutes and washed for 3 times. Each section was incubated with 50 *μ*l TUNEL detection mixture in the dark for 1 hour at 37°C. The sections were washed and then evaluated by a fluorescence microscope. Positive cells were measured by ImageJ software (National Institutes of Health, Bethesda, MD).

### 2.10. Immunofluorescence Analysis

Briefly, RAW264.7 cells (4 × 10^4^/well) were seeded on cover glass slips in 12-well plates and treated as described in Section 2.5. Then, cells were washed with PBS and then fixed in 4% paraformaldehyde for 20 min at room temperature. Then, cells were administrated with Triton X-100 (0.1%, 10 min) and blocked in 5% bull serum albumin (BSA) for 60 min. Then, the cells were incubated with NF-*κ*B p65 antibody (1 : 500) 10 h at 4°C. After washing, the second antibody was added and incubated in the dark for 60 min. Finally, DAPI was added for 5 min to display the nucleus.

### 2.11. Measurements of Reactive Oxygen Species

Reactive oxygen species (ROS) was measured with a DCFH-DA probe as we described before [16]. Briefly, NCTC 1469 cells were treated with 10 *μ*M DCFH-DA for 45 min. Then, inflorescence was recorded by a FACSCanto II flow cytometer (BD Biosciences, San Jose, CA, USA). Data were analyzed using FCS express 3 (De Novo Software). The ROS levels were expressed as mean fluorescence intensity (MFI).

### 2.12. RNA Interference

RAW264.7 and NCTC 1469 were grown in 6-well plates to 40-50% confluent. Then, 50 nM Nrf2 siRNA or control siRNA was transfected with Lipofectamine 6000 as we reported before [22]. The cells were used for the next experiments 48 h after transfection.

### 2.13. Western Blot

The proteins of hepatic tissues and cell lines were extracted by using the cell lysis buffer for western blot and IP (Beyotime Biotechnology) according to the producer's protocol. Protein concentration was measured by BCA Protein Assay Kit (Beyotime Biotechnology) according to the producer's protocol. Sodium dodecyl sulfate-polyacrylamide gel electrophoresis and immunoblotting were performed as we reported before with minor alteration [23]. Briefly, an equal amount of 20 *μ*g protein was loaded and then electron transferred onto PVDF membrane. The membrane was blocked in 5% skimmed mild for 1 h and then incubated with primary antibodies against Bax (1 : 1000), Bcl-2 (1 : 1000), NF-*κ*B p65 (1 : 1000), phosphor-NF-*κ*B p65 (1 : 1000), cleaved-PARP (1 : 1000), I*κ*B-*α* (1 : 1000), and GAPDH (1 : 1500) for overnight incubation. Then, the membrane was incubated with secondary antibodies conjugated with horseradish peroxidase (HRP) for 1 hour. Data were recorded on the Image Lab system and analyzed using ImageJ software.

### 2.14. Annexin V/7-Amino-Actinomycin D Staining

APC Annexin V Apoptosis Detection Kit with 7-AAD (BioLegend, San Diego, USA) was used to evaluate NCTC 1469 apoptosis strictly according to the producer's protocol. The stained cells were analyzed using a FACSCanto II flow cytometer (BD Biosciences, San Jose, CA, USA).

### 2.15. Statistical Analyses

The results were expressed as the mean ± standard deviation. GraphPad Prism 8 was used for statistical analyses. *p* value was calculated by one-way analysis of variance and followed by Tukey's post hoc test. *p* < 0.05 was considered significant.

## 3. Results

### 3.1. OI Improved the Survival Rate in LPS/D-GalN-Induced ALF Model

**Figure 1:**
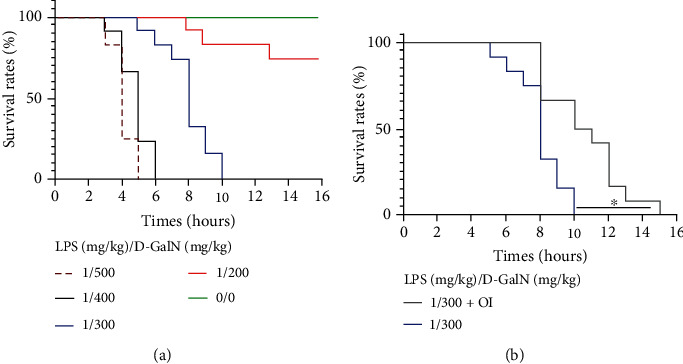
Improvement of survival rate in LPS/D-GalN-treated mice by OI. (a) Survival rates of the mice in different doses of LPS/D-GalN administration. (b) OI treatment improved the survival rate of LPS- (1 mg/kg)/D-GalN- (300 mg/kg) treated mice. (^∗^*p* < 0.05, *n* = 6-12); “ns” indicates not significant (*p* > 0.05).

To explore whether OI could improve the survival rate in LPS/D-GalN-induced ALF model, first, we used different doses of LPS/D-GalN to treat mice to determine the suitable survival rate. Survival was recorded for 16 hours. As shown in Figure 1(a), all mice died no more than 6 h in LPS (1 mg/kg)/D-GalN (400 mg/kg) and LPS (1 mg/kg)/D-GalN (500 mg/kg) groups. And mice died in 10 h in an intermediated dose of LPS (1 mg/kg)/D-GalN (300 mg/kg) group. The high doses of LPS/D-GalN induced too rapid progress of disease to establish the pathological process of the ALF model. Therefore, we chose a dose of LPS (1 mg/kg)/D-GalN (300 mg/kg) in our next experiment. As shown in Figure 1(b), the administration of OI significantly extended the median survival in the murine ALF model.

### 3.2. OI Mitigated LPS/D-GalN-Induced Histopathology and Liver Damage

**Figure 2:**
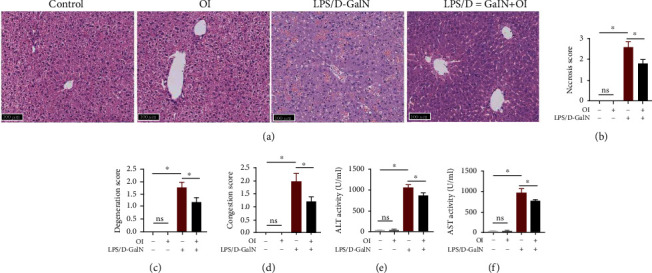
Treatment with OI mitigated LPS/D-GalN-induced hepatic injury. (a) Treatment of OI on LPS/D-GalN-induced changes in liver histopathology (200x) and the necrosis score (b), degeneration score (c), and congestion score (d). Serum ALT (e) and AST (f) activities of each group. Data are expressed as mean ± SEM (*n* = 5 for each group); (^∗^*p* < 0.05, *n* = 5). “ns” indicates not significant (*p* > 0.05).

As shown in Figure 2(a), histological examination showed no hepatocyte necrosis or cellular architecture damage in the control and OI groups. In contrast, LPS/D-GalN administration induced hepatic damage such as necrosis, degeneration, and congestion. However, OI treatment significantly attenuated these indices of hepatic injury (Figures 2(a)–2(d)). Moreover, ALT and AST are widely used as biomarkers to evaluate hepatic damage. As shown in Figures 2(e) and 2(f), LPS/D-GalN could markedly increase the serum activities of ALT and AST. However, the serum activities of ALT and AST were dramatically reduced in the LPS/D-GalN+OI group.

### 3.3. OI Inhibited Proinflammatory Cytokine Production

**Figure 3:**
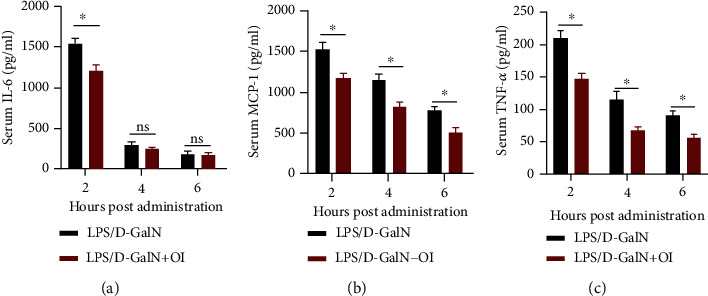
Effect of OI on activities of serum cytokines in LPS/D-GalN-treated mice. Serum levels of IL-6 (a), MCP-1 (b), and TNF-*α* (c) of different hours (2 h, 4 h, and 6 h) after LPS/D-GalN administration with or without OI treatment. Data are expressed as mean ± SEM (*n* = 5 for each group); (^∗^*p* < 0.05, *n* = 5). “ns” indicates not significant (*p* > 0.05).

Since proinflammatory mediators play pivotal roles in LPS/D-GalN-induced hepatic damage, we measured serum levels of TNF-*α*, IL-6, and MCP-1 in mice treated with LPS/D-GalN with or without OI at 2 h, 4 h, and 6 h after LPS/D-GalN administration. We found that LPS/D-GalN could increase TNF-*α*, IL-6, and MCP-1 expression in serum after LPS/D-GalN treatment. The TNF-*α*, IL-6, and MCP-1 levels reached the highest level after LPS/D-GalN treatment for 2 h and then gradually reduced from 2 h to 6 h. And OI treatment could significantly decrease serum TNF-*α*, IL-6, and MCP-1 levels in LPS/GalN-treated mice (Figures 3(a)–3(c)).

### 3.4. Effect of OI on Oxidative Stress and MPO Activity in LPS/D-GalN-Treated Mice

**Figure 4:**
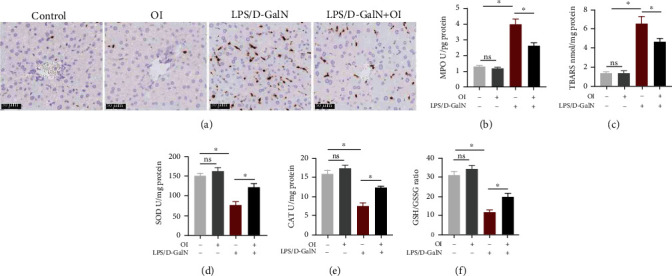
Effect of OI on MPO level and oxidative stress in LPS/D-GalN-treated mice. (a) Immunohistochemical assay of MPO-positive cells in hepatic sections of each group. (b) Activities of MPO in each hepatic tissues. (c) Levels of TBARS in liver tissues in each group. (d) Activities of SOD in liver tissues in each group. (e) Activities of CAT in each group. (f) The GSH/GSSG ratio was evaluated in each group. Data are expressed as mean ± SEM (*n* = 5 for each group); (^∗^*p* < 0.05, *n* = 5). “ns” indicates not significant (*p* > 0.05).

MPO is considered as a marker of neutrophils and an index of oxidative stress. We found that MPO activity and MPO-positive cells were elevated in LPS/D-GalN-treated mice. However, OI reduced MPO levels in LPS/D-GalN-treated mice (Figures 4(a) and 4(b)). To evaluate the oxidative stress in hepatic tissues, we measured the values of SOD, TBARS, CAT, and GSH/GSSH ratio. We found that the administration of LPS/D-GalN resulted in an elevation of TBARS content compared to that in the control group. And OI treatment markedly decreased the TBARS content (Figure 4(c)). Furthermore, LPS/D-GalN administration decreased levels of antioxidative enzymes including SOD and CAT while OI treatment could significantly restore SOD (Figure 4(d)) and CAT (Figure 4(e)) levels. Moreover, GSH/GSSG ratio represented the antioxidant capacity. In our results, GSH/GSSG ratio dramatically decreased in the LPS/D-GalN group compared to control. However, the administration of OI significantly increased the GSH/GSSG ratio (Figure 4(f)).

### 3.5. OI Mitigated LPS/D-GalN-Induced Apoptosis of Hepatocytes

**Figure 5:**
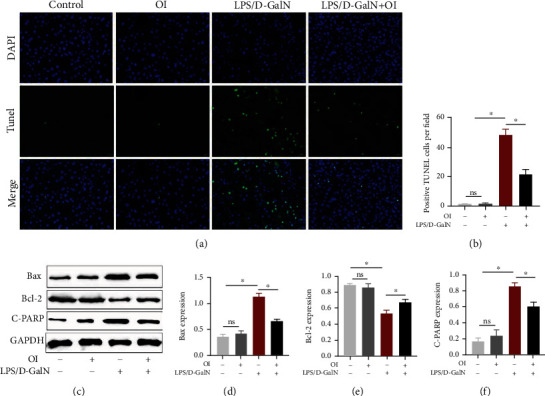
OI reduced LPS/D-GalN-induced cell death in liver. (a) Cell apoptosis was evaluated by TUNEL assay in hepatic tissue, and green fluorescence positive cells (b) were quantified in each group. (c–f) The Bax, Bcl-2, and cleaved-PARP levels were evaluated in each group. *Gapdh* was used as endogenous control. Data are expressed as mean ± SEM (*n* = 5 for each group); (^∗^*p* < 0.05, *n* = 5). “ns” indicates not significant (*p* > 0.05).

TUNEL assay was used to evaluate apoptosis of hepatocytes in hepatic tissues. We found that the cells' death markedly increased in the LPS/D-GalN group compared with the control group (Figures 5(a) and 5(b)). However, treatment of OI significantly diminished the TUNEL-positive cells in LPS/D-GalN-treated liver tissue. Single OI treatment had no effect on apoptosis. In addition, we measured the expression of Bax, Bcl-2, and cleaved-PARP in hepatic tissues (Figure 5(c)). Our results showed that LPS/D-GalN increased Bax (Figure 5(d)) and cleaved-PARP (Figure 5(f)) expression compared with the control group but decreased Bcl-2 (Figure 5(e)) expression in the liver. Compared with the LPS/D-GalN group, Bax and cleaved-PARP expression was decreased, while Bcl-2 was increased in the LPS/D-GalN+OI group. These indicated that OI reduced apoptosis in the liver.

### 3.6. OI Enhanced Nrf2 Expression and Inhibited NF-*κ*B Nuclear Translocation in LPS-Stimulated Macrophage

**Figure 6:**
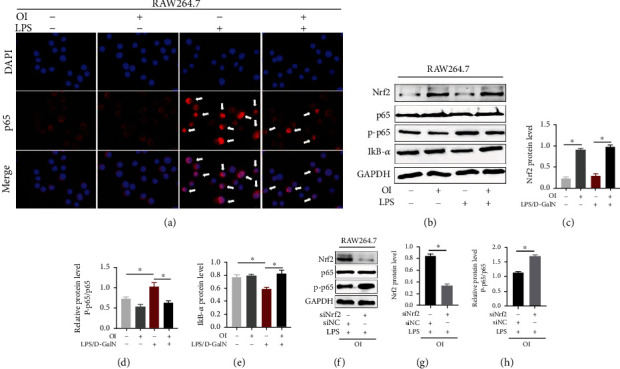
OI inhibited lipopolysaccharide-induced NF-*κ*B activation by increasing the expression of Nrf2. (a) Immunofluorescence assay to show NF-*κ*B nuclear translocation in RAW264.7 in each group. Arrows were used to mark the nuclear translocation. (b) Immunoblots of Nrf2, NF-*κ*B p 65, phosphate-p65 (p-p65), and I*κ*B-*α* in RAW264.7 in each group. And analysis for expression of Nrf2 (c), NF-*κ*B p-p65/p65 ratio (d), and I*κ*B-*α* (e) in each group. (f) Immunoblots of Nrf2, NF-*κ*B p65, phosphate-p65 (p-p65) in LPS- and OI-treated RAW264.7 macrophage with siRNA control (siNC) or siRNA *Nrf2* (siNrf2) treatment and analysis for expression of Nrf2 (g) and NF-*κ*B p-p65/p65 ratio (h). *Gapdh* was used for endogenous control. Data are expressed as mean ± SEM. All experiments were performed three times independently. (^∗^*p* < 0.05).

Macrophage activation aggravates LPS/D-GalN-induced liver injury. NF-*κ*B p65 phosphorylation and nuclear translocation are hallmarks of macrophage activation. Here, we used the murine macrophage cell line, RAW264.7, to explore the roles of OI in macrophages. As shown in Figure 6(a), LPS administration markedly increased p65 nuclear translocation compared with control and single OI groups. However, OI treatment reduced p65 nuclear translocation (Figure 6(a)). In addition, we measured the expression of Nrf2, NF-*κ*B p65, phospho-NF-*κ*B p65 (p-p65), and I*κ*B-*α* in RAW264.7 (Figure 6(b)). Our results showed that Nrf2 expression was elevated by OI (Figure 6(c)). Relative NF-*κ*B p-p65/p65 protein ratio was increased with LPS administration compared with the control group, and OI treatment could markedly reduce this elevation (Figure 6(d)). Accordingly, I*κ*B-*α* level was deceased by LPS, while OI reduced such reduction (Figure 6(e)). Moreover, we further explored the possible mechanisms by inhibiting Nrf2 expression by specifying siRNA (Figure 6(f)). After the expression of Nrf2 was inhibited (Figure 6(g)), we found that the relative NF-*κ*B p-p65/p65 protein ratio significantly increased (Figure 6(h)).

### 3.7. OI Reduced D-GalN-Induced Oxidative Stress and Apoptosis in NCTC 1469 Hepatocytes

**Figure 7:**
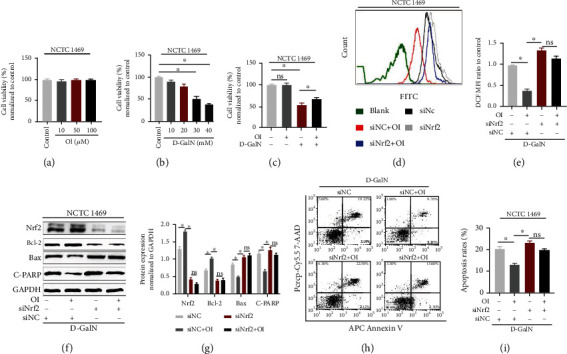
OI mitigated D-GalN-induced hepatocyte apoptosis by inhibiting oxidative stress via increasing Nrf2 expression. (a) Cell viability of NCTC 1469 murine hepatocytes treated with different concentrations of OI (10 *μ*M, 50 *μ*M, and 100 *μ*M). (b) Cell viability of hepatocytes treated with different concentrations of D-GalN (10 mM, 20 mM, 30 mM, and 40 mM). (c) Cell viability measured by CCK-8 assay in D-GalN-treated cells with or without OI. (d) Histogram of DCF FITC fluorescence. (e) Mean fluorescence intensity (MFI) of DCF was analyzed in each group. (f, g) Immunoblots of Nrf2, Bax, Bcl-2, and cleaved-PARP in D-GalN- and/or OI-treated hepatocytes with siRNA control (siNC) or siRNA *Nrf2* (siNrf2) treatment and analysis for expression of Nrf2, Bax, Bcl-2, and cleaved-PARP. *Gapdh* was used for endogenous control. (h, i) The apoptosis rates in D-GalN- and/or OI-treated hepatocytes with siRNA control (siNC) or siRNA *Nrf2* (siNrf2) treatment. Data are expressed as mean ± SEM. All experiments were performed three times independently. (^∗^*p* < 0.05). “ns” indicates not significant (*p* > 0.05).

D-GalN is a common chemical used for causing hepatic damage. We first tested whether OI alone could lead to hepatotoxic damage. Our results showed that a single treatment of OI (10 *μ*M, 50 *μ*M, and 100 *μ*M) had no statistical difference on the viability of NCTC 1469 compared to control (Figure 7(a)). Thus, we used 100 *μ*M concentration in the next experiment. Then, we measured the cell viability treated by different concentrations of D-GalN (10 mM, 20 mM, 30 mM, and 40 mM) to determine a suitable concentration for the next experiments. Concentration of 30 mM D-GalN treatment exhibited a viability of close to 50%; thus, this concentration was used in the next experiments (Figure 7(b)). And we found that OI treatment attenuated D-GalN-induced reduction of cell viability (Figure 7(c)). To further explore the underlying mechanism, we inhibited Nrf2 expression (Figure 7(f)) and then measured the ROS generation, apoptotic protein expression (Bax, Bcl-2, and cleaved-PARP), and cells' apoptosis in D-GalN-treated hepatocytes with or without OI treatment. We found that OI could inhibit ROS production induced by D-GalN (Figures 7(d) and 7(e)). However, deleting Nrf2 by siRNA eliminated this effect. Moreover, the antiapoptotic protein Bcl-2 was induced by OI, while this was eliminated with Nrf2 inhibition. Accordingly, the expression of Bax and c-PARP was inhibited by OI in D-GalN-treated cells. However, this effect was markedly inhibited by Nrf2 deletion (Figures 7(f) and 7(g)). We also evaluated the apoptosis rates by flow cytometry in hepatocytes. D-GalN significantly increased apoptosis. And OI could significantly reduce cell death in cells treated with D-GalN. However, this decreasing apoptotic effect by OI was markedly eliminated through inhibition of Nrf2 (Figures 7(h) and 7(i)).

## 4. Discussion

Severe or persistent liver injury will eventually lead to acute severe hepatitis or fulminant hepatitis, which is the major cause of death. LPS/D-GalN-induced liver injury is very similar to fulminant hepatic failure in humans. Therefore, this model was frequently used to screen hepatic protecting drugs. Itaconate, as a metabolite of the tricarboxylic acid cycle, recently showed protective properties in many pathologies by regulating inflammatory response and oxidative stress [18]. Additionally, OI, a derivate of itaconate, showed a protective effect in the hepatic ischemia-reperfusion injury murine model [12]. Moreover, our previous publication showed that OI protected mice from carbon tetrachloride-induced hepatic injury [22]. Nonetheless, the mechanism of liver injury is different in different pathogenic models. And the effect of OI in LPS/D-GalN-induced ALF has not yet been investigated. In our current study, we first found that OI attenuated LPS/D-GalN-induced liver damage and improved survival in the murine ALF model. Moreover, the inflammatory response and oxidative stress were mitigated by OI. These indicated the protective effect of OI in LPS/D-GalN-induced ALF.

Excessive inflammatory response is involved in LPS/D-GalN-induced hepatic damage [24]. In the model of LPS/D-GalN-induced ALF, LPS plays pivotal roles in pathogenesis by activating macrophages and other immune cells to produce many proinflammatory cytokines including TNF-*α*, IL-6, and MCP-1. TNF-*α* is an important cytokine in the pathogenesis of ALF. It could activate an inflammatory cascade to produce other proinflammatory cytokines and many adhesion molecules. TNF-*α* also plays a central role in hepatocytes apoptosis [17]. Previous report has validated that lacking expression of TNF-*α* and its receptor was tolerant to LPS/D-GalN-induced ALF [4]. In our current study, we observed many apoptotic cells in LPS/D-GalN-treated liver tissues by TUNEL assay. OI treatment markedly reduced these positive cells in LPS/D-GalN-intoxicated animal liver sections. Mechanically, TNF-*α* could activate the caspase cascade and initiate the apoptotic pathway [25]. At least in part, the activated caspase form like cleaved caspase-3 could cleave the PARP to produce cleaved-PARP which is validated as a crucial molecule to induce apoptosis [26]. Therefore, inhibition of TNF-*α* and other proinflammatory mediators by OI is crucial in alleviating ALF induced by LPS/D-GalN. Moreover, Bcl-2 family proteins are associated the LPS/D-GalN-induced apoptosis [3]. Bcl-2 family includes two functional different-type proteins, antiapoptotic type (such as Bcl-2) and proapoptotic type (such as Bax) [27]. The level of Bcl-2 and Bax determines the fate of the cell after apoptotic stimulus. Our results showed that OI inhibited apoptosis by increasing Bcl-2 expression and diminishing Bax expression in LPS/D-GalN-treated liver tissues.

In addition to inflammation, oxidative stress plays an important role in hepatic damage [9]. Normally, the antioxidative defense system can eliminate ROS to avoid excessive oxidative stress [28, 29]. LPS/D-GalN administration led to excessive production of free radicals and caused loss of antioxidant capacity in the liver, which brought oxidative stress and lipid peroxidation [30]. And lipid peroxidation will eventually cause cell death [31, 32]. TBARS level is widely used to evaluate extent of lipid peroxidation and oxidative stress. Apart from ROS, MPO is another marker that may reflect oxidative stress. MPO was not only considered as a marker of neutrophils but also could produce hypochlorite by hydrogen peroxide and chloride ions to form free radicals, which may lead to oxidative stress and tissue injury [33]. In our study, we found that OI markedly diminished levels of TBARS and MPO in a LPS/D-GalN-treated model. Meanwhile, enzymatic and nonenzymatic antioxidant systems play pivotal roles in alleviating oxidation-associated damage. A previous study has confirmed that antioxidant enzymes like SOD and CAT were decreased upon LPS/D-GalN administration [34]. Moreover, LPS/D-GalN administration also reduced the content of reduced thiol group like GSH [34, 35]. Our results also indicated a decrease of the activities of SOD and CAT and GSH/GSSG ratio upon LPS/D-GalN treatment, which is parallel with the former reports. However, OI administration significantly restored SOD and CAT activities and GSH/GSSG ratio. Therefore, our results revealed that the antioxidant property of OI was associated with the protective effect against LPS/D-GalN-induced hepatic injury.

We further explored the possible mechanism of OI's protective effect in LPS/D-GalN-induced hepatic injury. For one thing, hepatic macrophages are critical in the enhancement of inflammation by overexpression of proinflammatory cytokines, which may lead to damage to liver tissues [36]. LPS could induce hepatic damage as it could activate macrophages by activating NF-*κ*B signaling and bring excessive inflammatory response [37] . NF-*κ*B activation was triggered by degradation of I*κ*B-*α* and NF-*κ*B translocation into the nucleus [15]. Then, NF-*κ*B transcription factors bind to corresponding sites and regulated procytokines' transcription. Phosphorylation of NF-*κ*B p65 was reported to enhance transcription effect [38]. Inhibition of NF-*κ*B signaling could reduce production of many proinflammatory cytokines [39]. Here, we found that OI could significantly inhibit LPS-induced NF-*κ*B p65 nuclear translocation in murine macrophages (Figure 6(a)). Meanwhile, OI enhanced the expression of Nrf2 and reduced the elevation of NF-*κ*B p-p65/p65 ratio induced by LPS in RAW264.7 (Figure 6(b)). Moreover, LPS-induced I*κ*B-*α* degradation was inhibited by OI (Figure 6(e)). Previous report found that OI could activate Nrf2 signaling [19]. To further explore the reason of NF-*κ*B inhibition, Nrf2 was inhibited by siRNA in macrophages. Then, our results showed that LPS-induced elevation of NF-*κ*B p-p65/p65 ratio was not inhibited by OI on a condition of Nrf2 deletion. Therefore, OI inhibited the inflammatory response in macrophages by activating Nrf2 to inhibit NF-*κ*B activation. For another, D-GalN can cause hepatotoxicity by depletion of GSH and interfered the synthesis of RNA [40]. D-GalN alone could induce the production of ROS in human HepG2 cells and L02 cells and lead to oxidative stress in hepatocytes [41]. ROS generation could affect mitochondrial membrane potential to initiate apoptosis in the mitochondrial pathway [42]. Normally, mitochondrial-associated apoptosis is involved in releasing cytochrome c, expression of Bax, inhibition of Bcl-2, and cleaving PARP. It was validated that excessive ROS could lead to hepatocyte apoptosis and D-GalN-induced apoptosis was also involved in this ROS-related pathway [41]. In our experiment, we found that D-GalN reduced the viability and caused ROS accumulation and apoptosis in NCTC 1469 cells (Figure 7). Nrf2 is a critical transcriptional nuclear factor that regulates many antioxidative genes. ROS could be eliminated, and oxidative stress was mitigated by activating Nrf2 signaling [43]. Our results showed that OI, as an activator of Nrf2, partially restored the viability of NCTC 1469 treated by D-GalN. Moreover, we found that OI reduced oxidative stress and subsequent apoptosis in D-GalN-treated NCTC 1469 cells and this protective effect was associated with Nrf2 activation (Figure 7).

## 5. Conclusion

Our current study showed that OI protected mice from LPS/D-GalN-induced ALF by inhibiting inflammatory response, oxidative stress, and apoptosis. The possible mechanism, at least in part, is associated with the activation of Nrf2 signaling in macrophages and hepatocytes. On the one hand, in macrophages, OI inhibited NF-*κ*B activation by activating Nrf2 to repress excessive inflammation which may lead to hepatocytes damage. On the other hand, this protective effect was associated with reducing oxidative stress and apoptosis mediated by ROS in hepatocytes. Therefore, our results revealed the effect of OI in LPS/D-GalN-induced ALF and provided a theoretical basis as a prevention for ALF.

## Data Availability

The data used to support the findings of this study are available from the corresponding author upon request.
